# Temporarily Out of Order: Temporal Perspective Taking in Language in Children With Autism Spectrum Disorder

**DOI:** 10.3389/fpsyg.2018.01663

**Published:** 2018-09-05

**Authors:** Jessica Overweg, Catharina A. Hartman, Petra Hendriks

**Affiliations:** ^1^Center for Language and Cognition Groningen (CLCG), University of Groningen, Groningen, Netherlands; ^2^Department of Psychiatry, University Medical Center Groningen, Groningen, Netherlands

**Keywords:** autism spectrum disorder, executive functioning, perspective taking, temporal conjunctions, Theory of Mind

## Abstract

Clinical reports suggest that children with autism spectrum disorder (ASD) struggle with time perception, but few studies have investigated this. This is the first study to examine these children’s understanding of *before* and *after*. These temporal conjunctions have been argued to require additional cognitive effort when conjoining two events in a clause order that is incongruent with their order in time. Given the suggested time perception impairment and well-established cognitive deficits of children with ASD, we expected them to have difficulties interpreting temporal conjunctions, especially in an incongruent order. To investigate this, the interpretation of *before* and *after* in congruent and incongruent orders was examined in 48 children with ASD and 43 typically developing (TD) children (age 6–12). Additional tasks were administered to measure Theory of Mind (ToM), working memory (WM), cognitive inhibition, cognitive flexibility, IQ, and verbal ability. We found that children with ASD were less accurate in their interpretation of temporal conjunctions than their TD peers. Contrary to our expectations, they did not have particular difficulties in an incongruent order. Furthermore, older children showed better overall performance than younger children. The difference between children with ASD and TD children was explained by WM, ToM, IQ, and verbal ability, but not by cognitive inhibition and flexibility. These cognitive functions are more likely to be impaired in children with ASD than in TD children, which could account for their poorer performance. Thus, the cognitive factors found to affect the interpretation of temporal language in children with ASD are likely to apply in typical development as well. Sufficient WM capacity and verbal ability may help children to process complex sentences conjoined by a temporal conjunction. Additionally, ToM understanding was found to be related to children’s interpretation of temporal conjunctions in an incongruent order, indicating that perspective taking is required when events are presented out of order. We conclude from this that perspective-taking abilities are needed for the interpretation of temporal conjunctions, either to shift one’s own perspective as a hearer to another point in time, or to shift to the perspective of the speaker to consider the speaker’s linguistic choices.

## Introduction

Time is an important dimension by which we make sense of the world (Navon, 1978). Time is also deeply rooted in the structural organization of language (Klein, 1994). In language, time is generally conceived as a sequential order of events, where one event follows another from past to present to future. Speakers can use temporal expressions, like *before* or *after*, to express the order of events in time either in order of occurrence (i.e., temporally congruent) or out of order (i.e., temporally incongruent). The interpretation of the temporal conjunctions *before* and *after* in an incongruent order is found to be difficult for typically developing (TD) children (Clark, 1971; Pyykkönen and Järvikivi, 2012; Blything et al., 2015; de Ruiter et al., 2018). This may hold even more for children with an autism spectrum disorder (ASD). Clinical reports suggest that children with ASD encounter difficulties in time perception (Wing, 1996). Additionally, some studies have suggested that individuals with ASD have difficulty interpreting *before* and *after* (Boucher, 2001; Perkins et al., 2006). The present study investigates time perception in language in children with ASD and their TD peers by examining their interpretation of sentences containing temporal conjunctions.

*Before* and *after* are viewed as the prototypical linguistic expressions indicating temporal order (Schilder and Tenbrink, 2001). Speakers can use these expressions in several ways to express the order of events. For example, all four sentences below indicate that someone first climbed a tree and next read a book:

(1) He climbed the tree before he read the book.
(2) Before he read the book, he climbed the tree.
(3) He read the book after he climbed the tree.
(4) After he climbed the tree, he read the book.

The speaker’s choice of *before* in a main-subordinate clause order (1) and *after* in a reversed clause order (4) result in a congruent presentation of the temporal order of events, whereas *before* in a subordinate-main clause order (2) and *after* in a reversed clause order (3) result in an incongruent presentation. Thus, it depends on the speaker’s choice of type of conjunction and clause order whether the hearer should interpret the event order as congruent or incongruent.

Developmental studies in TD children report that congruency has an effect on the correct interpretation of *before* and *after* (Clark, 1971; Trosborg, 1982; McCormack and Hanley, 2011; Pyykkönen and Järvikivi, 2012; Blything et al., 2015; de Ruiter et al., 2018). Children under the age of 7 have more difficulties interpreting conjunctions in a temporally incongruent order than in a temporally congruent order, and mostly rely on the order of presentation of the events. Pyykkönen and Järvikivi (2012) showed that children between 8 and 12 years old still experience difficulties interpreting temporal conjunctions in an incongruent order, especially when the cue to event order occurs sentence-medially, as in example sentence (3).

Children’s difficulties with interpreting temporal conjunctions in an incongruent order have been explained in various ways. For example, these difficulties have been argued to result from a still fragile understanding of the meaning of the temporal conjunctions *before* and *after* (Clark, 1971), from difficulty shifting one’s perspective to a different point in time (McCormack and Hoerl, 1999; McCormack and Hanley, 2011), from difficulty processing subordinate-main clause orders (Diessel, 2008), and from difficulty holding information active in working memory (WM) during processing to create a chronological mental representation of the events (Blything et al., 2015; Blything and Cain, 2016). In adults, interpreting temporal conjunctions in an incongruent rather than congruent order comes with processing costs and has been shown to tax WM (Münte et al., 1998). So, interpreting temporal conjunctions in an incongruent order may require additional cognitive effort.

According to anecdotal evidence and clinical reports, individuals with ASD encounter difficulties in time perception (Wing, 1996). They often report a need to adhere to rituals and routines and are commonly preoccupied with timetables, clocks, and calendars, which may serve to compensate for their failure to predict future events and their disorientation in time (Allman and DeLeon, 2009). This led Boucher (2001) to suggest that individuals with ASD have an impaired sense of time. So far, few studies have been conducted on time perception in children with ASD. Some studies report intact time perception (Wallace and Happé, 2008; Gil et al., 2012), while other studies suggest that children with ASD experience particular difficulties with understanding temporal ordering and concepts such as duration, succession, past, and future (Gillberg and Peeters, 1995; Boucher et al., 2007; Maister and Plaisted-Grant, 2011). Also, some studies report that children with ASD use fewer temporal expressions in story-telling (Colle et al., 2008) and more often omit tense marking than their TD peers (Roberts et al., 2004). These findings regarding the production of temporal expressions suggest that children with ASD may struggle with their interpretation of temporal conjunctions as well, although a mismatch between their production abilities and their comprehension abilities is also conceivable (see Hendriks, 2014 for an overview and discussion of attested production–comprehension asymmetries in child language).

Executive functioning (EF) impairments, often present in children with ASD (Hill, 2004), could make it especially difficult to interpret temporal conjunctions in an incongruent order. EF refers to cognitive processes such as WM (the capacity system that allows the temporary storage and manipulation of information necessary for complex tasks such as language comprehension; Baddeley, 2000), inhibition (the mental ability to suppress irrelevant information; Dagenbach and Carr, 1994), and flexibility (the mental ability to shift between different thoughts or actions; Scott, 1962), that allow for the flexible alteration of thought and behavior in response to changing contexts (Welsh and Pennington, 1988). Recent studies have argued that TD children between 3 and 7 years old have more difficulties interpreting temporal conjunctions in an incongruent order than in a congruent order because more information must be maintained in WM to revise the mental representation of the events and create a chronological mental representation (Blything et al., 2015; Blything and Cain, 2016). The neuroimaging studies of Münte et al. (1998) and Ye et al. (2012) suggest that, also for adults, WM is needed for the temporal re-ordering of events. Furthermore, the ability to inhibit an initial interpretation and to flexibly revise a mental representation of event order could be needed to interpret conjunctions in an incongruent order (Pyykkönen and Järvikivi, 2012; Blything and Cain, 2016). Thus, in addition to WM, also cognitive inhibition and cognitive flexibility may be involved.

In addition to impairments in these EF functions, also impairments in Theory of Mind (ToM) understanding (Frith and Frith, 2006) could make it difficult for children with ASD to interpret temporal conjunctions in an incongruent order. ToM is the ability to take the cognitive perspective of other people to understand their beliefs, desires and intentions (Wimmer and Perner, 1983) and is argued to be impaired in children with ASD (Baron-Cohen et al., 1985). If the interpretation of an incongruent temporal order involves ToM understanding, an incongruent temporal order may be especially difficult for children with ASD. Several studies have suggested that the interpretation of temporal language not only requires a consideration of the actual perspective in time but also a consideration of alternative temporal perspectives (McGlone and Harding, 1998; McCormack and Hoerl, 1999; Stocker, 2012). According to McCormack and Hoerl (1999), hearers should not only be able to shift from the actual perspective in time to alternative temporal perspectives, but should also understand the relation between these perspectives. Based on their account of the development of temporal understanding, they posit that “temporal perspective taking involves mentalizing abilities” (McCormack and Hoerl, 1999; p. 174). Thus, mentalizing, or ToM understanding, could be involved in the comprehension of an incongruent order of events.

This is the first study to investigate how 6- to 12-year-old children with ASD and their TD peers interpret temporal conjunctions. We expect that all children find the interpretation of *before* and *after* more difficult in the incongruent order than in the congruent order, but that children with ASD find the interpretation of these temporal conjunctions in an incongruent order more difficult than their TD peers. As EF and ToM have been reported to be possibly impaired in individuals with ASD, this may explain the hypothesized difficulties with the interpretation of temporal conjunctions in children with ASD. Therefore, we further hypothesize that differences in the interpretation of temporal conjunctions in an incongruent order are associated with individual differences in EF and ToM understanding. In addition to the specific cognitive factors EF and ToM, we also examine the role of the more general cognitive factors IQ and verbal ability. EF and ToM may not only provide insight into the individual differences in ASD that play a role in temporal language understanding, but may also provide insight into what it is in the broad measures of IQ and verbal ability that possibly explains temporal language understanding.

## Materials and Methods

### Participants

In this study, 48 children with ASD and 43 TD children participated. All children were monolingual native Dutch children who did not have any reported language disorders. The children in the ASD group were diagnosed with ASD by clinicians on the basis of the DSM-IV-TR criteria (American Psychiatric Association, 2000) and had an IQ of >75 based on a clinically administered full IQ test. Additionally, in all children (ASD as well as TD), certified professionals administered the Autism Diagnostic Observation Schedule (ADOS; Lord et al., 1999), the Autism Diagnostic Interview Revised (ADI-R; Rutter et al., 2003), two subtests (Vocabulary and Block Design) of the WISC-III-NL to estimate IQ (Kort et al., 2002), and the Peabody Picture Vocabulary Test to measure Verbal Ability (VA) (PPVT-III-NL; Schlichting, 2005). Two children from the ASD group were excluded because they neither met the ADOS criteria for ASD nor the ADI-R criteria for ASD (cf. Risi et al.’s., 2006, ASD2 criteria). One child from the TD group met the ADOS criteria for ASD and was therefore excluded as well, leaving 46 children with ASD (mean age = 9;4, SD = 2;2) and 42 TD children (mean age = 9;2, SD = 2;0) for further analysis. The group descriptives of the ASD group and the TD group are provided in **Table 1**.

**Table 1:** Description of the participants with autism spectrum disorder (ASD) and the typically developing (TD) participants in this study.

Background variables	ASD (*N* = 46)	TD (*N* = 42)	Group differences (general linear model ANOVA analyses)
Gender (boys:girls)	39:7	34:8	n.s.
**Chronological age (year; month)**			
Mean (*SD*)	9;4 (2;2)	9;2 (2;0)	n.s.
Range	6;0–12;5	6;2–12;7	
**Clinical diagnosis of ASD subtype according to DSM-IV criteria (*N*)**			
Autistic disorder	4	0	–
Asperger’s disorder	2	0	–
PDD-NOS^a^	40	0	–
**Number of participants meeting ASD2 criteria^b^ on**			
ADOS and ADI	33	0	–
ADOS only	10	1 (excluded)^e^	–
ADI only	3	0	–
Neither ADOS nor ADI	2 (excluded)^e^	42	–
**Estimated IQ (WISC)^c^**			
Mean (*SD*)	99.87 (16.92)	113.21 (13.86)	TD > ASD^∗∗∗^
Range	66.65–145.48	72.71–145.48	
**Verbal ability score (PPVT)^d^**			
Mean (*SD*)	104.48 (13.9)	113.62 (11.53)	TD > ASD^∗∗^
Range	77–139	87–138	

*^a^PDD-NOS: pervasive developmental disorder-not otherwise specified; ^b^The ASD2 criteria of Risi et al. (2006) are: “a child meets criteria on Social and Communication domains or meets criteria on Social and within two points of Communication criteria or meets criteria on Communication and within two points of Social criteria or within one point on both Social and Communication domains” (Risi et al., 2006; p. 1100); ^c^Estimated IQ of two subtests of the Dutch version of the Wechsler Intelligence Scale for Children (WISC-III-NL; Kort et al., 2002); ^d^Normed verbal ability score from the Dutch version of the Peabody Picture Vocabulary Test (PPVT-III-NL; ^e^Excluded from the group descriptives in this table as well as from the analyses; Schlichting, 2005); ^∗∗^p < 0.01; ^∗∗∗^p < 0.001.*

Children with ASD were recruited *via* outpatient clinics for child and adolescent psychiatry in Groningen and a national website for parents with children with ASD. TD children were recruited *via* advertising in newsletters and flyers at schools in the north of Netherlands. The children were tested individually on a single day in a quiet room at the university with two experimenters present. This study is part of a wider study on language and perspective taking in children with ASD, in which all children of the current study participated. The medical ethical committee of the University Medical Hospital Groningen evaluated this study as not falling under the Medical Research Involving Human Subjects Act (WMO). Nevertheless, we followed the required procedures and obtained written informed consent from the parents of all participants for their child’s participation in the research.

### Language Comprehension Task

**FIGURE 1:**
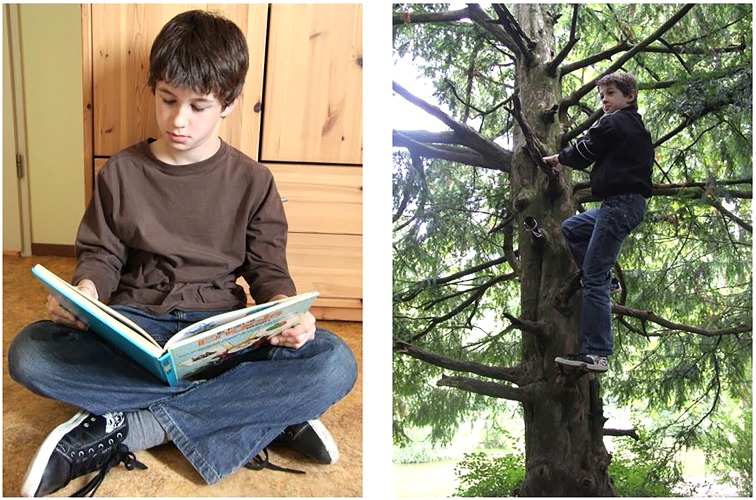
An example of the two pictures of an item in the language comprehension task. Written informed consent was obtained from the parents for publication of their child’s images.

Comprehension of temporal conjunctions was tested using a picture selection task. Per item, participants saw two pictures side by side on a computer screen, each depicting an event (see **Figure 1**).

Simultaneously, they heard a pre-recorded sentence describing the temporal order of the two events. Participants had to press one of two buttons on a button box to select the picture that, according to the sentence, showed the event that happened first. The sentences contained either *voordat* (“before”) or *nadat* (“after”), which occurred either in sentence-initial position (corresponding to subordinate-main clause order) or in sentence-medial position (corresponding to main-subordinate clause order). Examples of each of the four conditions (conjunction × position) in the language comprehension task are shown below in Dutch, followed by word by word glosses and English translations:

(1) *voordat* (“before”) in sentence-initial position:
Voordat hij het boek las, klom hij in de boom.
before he the book read, climbed he in the tree
“Before he read the book, he climbed the tree.”
(2) *voordat* (“before”) in sentence-medial position:
Hij klom in de boom voordat hij het boek las.
he climbed in the tree before he the book read
“He climbed the tree before he read the book.”
(3) *nadat* (“after”) in sentence-initial position:
Nadat hij in de boom klom, las hij het boek.
after he in the tree climbed, read he the book
“After he climbed the tree, he read the book.”
(4) *nadat* (“after”) in sentence-medial position:
Hij las het boek nadat hij in de boom klom.
he read the book after he in the tree climbed
“He read the book after he climbed the tree.”

The events in sentences (2) and (3) are mentioned in a congruent order, whereas the events in (1) and (4) are mentioned in an incongruent order. All events were unrelated to avoid a preference for one of the two event orders based on event typicality.

Stimuli were presented and responses were recorded using the computer software E-Prime 2.0 (Schneider et al., 2002). First, children completed three practice items to practice that the left and right button corresponded to the left and right picture, respectively. This was followed by an introduction of the boy in the pictures and three practice items containing other temporal expressions (e.g., “today” and “yesterday”) to determine whether the participant understood the principle of temporal ordering in the task. Next, the participants received 32 test items, with a short break in the middle. The test items were distributed across 4 lists. Each list contained 16 congruent test items and 16 incongruent test items in a randomized order. We counterbalanced the position of the pictures on the screen. The experiment took approximately 15 min.

### Cognitive Tasks

#### Working Memory

To test WM, the N-Back task (Owen et al., 2005) was used. In this task, participants had to watch and remember pictures presented one by one on a computer screen and indicate whether the picture on the screen was a particular object or not (0-back or baseline condition), whether it matched the picture one trial before (one-back condition), and whether it matched the picture two trials before (two-back condition). Participants received a practice session of 15 trials per condition and a test session consisting of 60 trials per condition. The mean accuracy (ACC) on the two-back condition was calculated as a measure of WM.

#### Cognitive Inhibition

To test cognitive inhibition, the Flanker task [Amsterdam Neuropsychological Test battery (ANT) version 2.1; De Sonneville, 1999] was administered. In this task, participants had to identify the color of a target stimulus surrounded by eight distractors (flankers). The target color was red or green and was associated with the left or right button, respectively. The flankers were either in the same color as the target (compatible trials) or in the color that was associated with the opposite response (incompatible trials). For this task, participants received 12 practice items, 40 compatible test items, and 40 incompatible test items. The mean ACC and mean reaction time (RT) of cognitive inhibition was measured by subtracting the mean ACC or RT on compatible trials from the mean ACC or RT, respectively, on incompatible trials (resulting in the congruency effect; see Mullane et al., 2009).

#### Cognitive Flexibility

To test cognitive flexibility, we adapted the gender emotion switch task of De Vries and Geurts (2012) to make it more similar to a classical switch task (e.g., Rogers and Monsell, 1995). In our shape–color switch task, participants saw pictures of round or square figures in black or white on the computer screen and had to press the left or right button to report the shape (round or square) or the color (black or white) of the figure. The cue at the top of the screen indicated whether the shape or the color had to be reported. Participants received 16 items to practice with shape, 16 items to practice with color, and 40 items to practice with switching between shape and color. The test consisted of 216 trials in total; a third of these trials (72) were switch trials (switching from color to shape or *vice versa*) and the remaining two third were repeat trials. The mean ACC and mean RT of switch costs was measured by subtracting the mean ACC or RT on repeat trials from the mean ACC or RT, respectively, on switch trials (cf. De Vries and Geurts, 2012).

#### Theory of Mind

To test first-order and second-order ToM, the Bake Sale task adapted from Hollebrandse et al. (2014) was used. This task is a second-order false belief (FB) task with stories modeled after Perner and Wimmer’s (1985) “ice cream truck story” in which the beliefs of various characters were manipulated. Per story, participants heard a verbal description of the events in the story, accompanied by four pictures that were presented one by one. During the presentation of the story, they received three questions to probe their understanding of the events in the story, as well as a question about the FB of another person (first-order FB question) and a question about the FB of another person about a second person (second-order FB question). The task consisted of eight stories in total, each of which contained a first-order FB question and a second-order FB question. The measures of ToM1 and ToM2 were calculated using the ACC on the eight first-order FB questions and the ACC on the eight second-order FB questions, respectively.

### Data Analysis

The data of the language comprehension task were analyzed using generalized linear mixed models (GLMMs), using a logit link to accommodate the repeatedly measured (32 trials) binary outcome variable Accuracy (0 for incorrect, 1 for correct) (Jaeger, 2008; Heck et al., 2012). Compound symmetry was used as the covariance matrix type. We set out with a full factorial model with Congruency (Congruent vs. Incongruent) as within group factor and Group (TD vs. ASD) as between group factor. Age was mean-centered and additionally included in the model. Interactions that did not have an effect on Accuracy (*p* > 0.05) were removed from the model one by one, starting with the interaction with the largest *p*-value, after which we refitted the model. This resulted in model 1, which shows the extent to which Accuracy was predicted by Congruency, Group, and Age, as well as the relevant (*p* < 0.05) interactions. The possible presence of effects related to Type of conjunction (Before vs. After) and Clause order (Main-subordinate vs. Subordinate-main) were subsequently checked, *post hoc*, in model 1. For purposes of interpretation, we illustrate significant effects using the median split method.

Next, the seven parameters derived from the N-Back task (WM), the Flanker task (Cognitive inhibition ACC and Cognitive inhibition RT), the cognitive flexibility task (Switch costs ACC and Switch costs RT) and the FB task (ToM1 and ToM2) were mean-centered and, one by one, examined as main effects and in interaction with the significant predictors from model 1 in seven separate analyses. The data of 3 participants (2 ASD and 1 TD) were missing in the Cognitive inhibition ACC and RT analyses, leaving the data of 44 participants with ASD and 41 TD participants. In each separate analysis, interactions that had no effect on Accuracy (*p* > 0.05) were removed from the model. Based on the outcomes of these analyses per predictor, we combined the cognitive factors with (main or interaction) effects on Accuracy (*p* < 0.05) and added these with the significant predictors of model 1 in a model with multiple predictors to evaluate their effects adjusted for one another (cf. Kuijper et al., 2015; Overweg et al., 2018). This resulted in model 2, which shows the relevant cognitive factors that had an effect on the interpretation of temporal conjunctions.

Finally, the parameters from the WISC (estimated IQ on the basis of the subtests Vocabulary and Block Design) and PPVT (VA) were mean-centered and included in two separate analyses in model 1. If they had an effect on Accuracy (*p* < 0.05), they were added to model 2 and evaluated in model 3. This resulted in model 3, which shows whether these general background variables changed the effects found in model 2. Given the significant group differences (see **Table 1**) in estimated IQ and VA, this approach provides a statistical alternative to *a priori* matching on estimated IQ and VA.

## Results

Model 1 showed main effects of Group and Age, indicating that the children in the TD group were more accurate in their interpretation of temporal conjunctions than the children in the ASD group, and that the older the child was, the better its performance. No main effect or interactions with Congruency were found (all *p*-values >0.05). A *post hoc* exploration of Type of conjunction and Clause order in model 1 showed a main effect of Type of conjunction (*B* = -0.943; *SE* = 0.14; *p* = 0.00), indicating that children perform better on sentences with *before* than on sentences with *after*. Clause order did not influence performance (*p* > 0.05). **Table 2** lists all remaining effects in model 1.

**Table 2:** Estimated effects of variables per model on the interpretation of temporal conjunctions.

Variables	Models								
	Model 1			Model 2			Model 3		
	Estimate	SE	*p*	Estimate	SE	*p*	Estimate	SE	*p*
Intercept	1.873	0.194	0.00**	1.605	0.192	0.00**	1.608	0.195	0.00**
Group	-0.549	0.191	0.01*	-0.114	0.215	0.60	0.085	0.231	0.71
Age	0.025	0.004	0.00**	0.006	0.006	0.25	0.017	0.006	0.00**
Congruency	-0.292	0.188	0.12	-0.173	0.171	0.32	-0.169	0.174	0.33
ToM1	-	-	-	1.253	0.813	0.13	1.051	0.744	0.17
ToM2	-	-	-	-0.334	0.401	0.41	-0.698	0.420	0.10
WM	-	-	-	1.735	0.788	0.03*	1.204	0.722	0.10
ToM2*Congruency	-	-	-	1.502	0.567	0.01*	1.544	0.586	0.01*
IQ	-	-	-	-	-	-	0.015	0.006	0.02*
VA	-	-	-	-	-	-	0.016	0.007	0.03*
VA*Age	-	-	-	-	-	-	0.001	0.000	0.00**

*The models were built with accuracy in the language comprehension task as the dependent variable and the variables listed in the first column as independent variables. The variable Congruency was manipulated by Type of conjunction (Before vs. After) and Clause order (Main-subordinate vs. Subordinate-main), with Before+Main-subordinate and After+Subordinate-main resulting in Congruent items, and Before+Subordinate-main and After+Main-subordinate resulting in Incongruent items. A post hoc exploration of Type of conjunction and Clause order in model 1 showed a main effect of Type of conjunction (B = -0.943; SE = 0.14; p = 0.00); ^∗^p = < 0.05; ^∗∗^p = < 0.01.*

**FIGURE 2:**
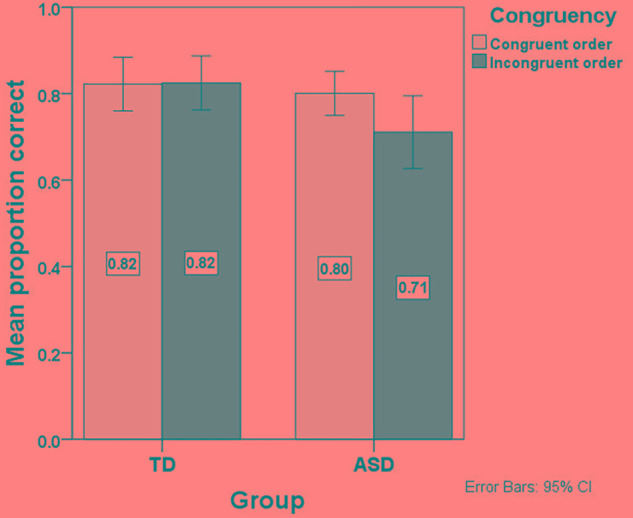
Mean proportion of correct responses in the language comprehension task per Congruency condition (Congruent vs. Incongruent) and Group (TD vs. ASD).

**Figure 2** presents the mean proportions of correct responses in the congruent and incongruent condition separately for the ASD and TD groups.

Next, we examined one by one which cognitive factors were associated with Accuracy. The separate analyses indicated a main effect of WM (*B* = 2.355; *SE* = 0.747; *p* = 0.002) and interactions of ToM1^∗^Congruency (*B* = 2.325; *SE* = 1.034; *p* = 0.026) and ToM2^∗^Congruency (*B* = 1.465; *SE* = 0.552; *p* = 0.009). No effects of Cognitive inhibition and Cognitive flexibility were found (*p*-values <0.05).

Then, we combined all significant interactions and main effects of these analyses per predictor in model 2, a model with multiple predictors. The interaction effect of ToM1^∗^Congruency was no longer significant when adjusted for the other cognitive variables and was removed from the model. **Table 2** lists all remaining effects in model 2.

**FIGURE 3:**
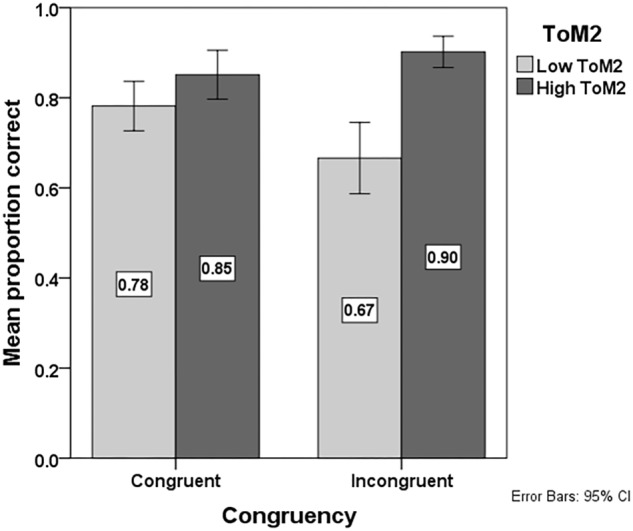
Mean proportion of correct responses in the language comprehension task per Congruency condition (Congruent vs. Incongruent) and second-order Theory of Mind (ToM2) group (low ToM2: ≤median vs. high ToM2: >median; median = 0.75). Background information about the two groups plotted in this figure regarding their ToM2 performance, as mean proportions of correct responses on the second-order false belief (FB) questions in the FB task: Low ToM2 group: 0.38; High ToM2 group: 0.94.

Model 2 showed a main effect of WM (*p* = 0.03; see **Table 2**), indicating that children with lower WM are less accurate in their interpretation of temporal conjunctions than children with higher WM. Model 2 also showed an interaction effect of ToM2^∗^Congruency (*p* = 0.01; see **Table 2**). As is shown in **Figure 3**, children with lower second-order ToM understanding are less accurate in their interpretation of temporal conjunctions in the Incongruent condition than children with higher second-order ToM understanding. The median split method is used to plot Accuracy of temporal conjunction interpretation in each condition per ToM2 group (low ToM2: ≤0.75 vs. high ToM2: >0.75) to illustrate the direction of the interaction effect. The figure caption of **Figure 3** provides background information about the ToM performance of each group.

The main effects of Group and Age disappeared with the addition of ToM2 and WM in model 2 (all *p*-values >0.05; see **Table 2**).

**FIGURE 4:**
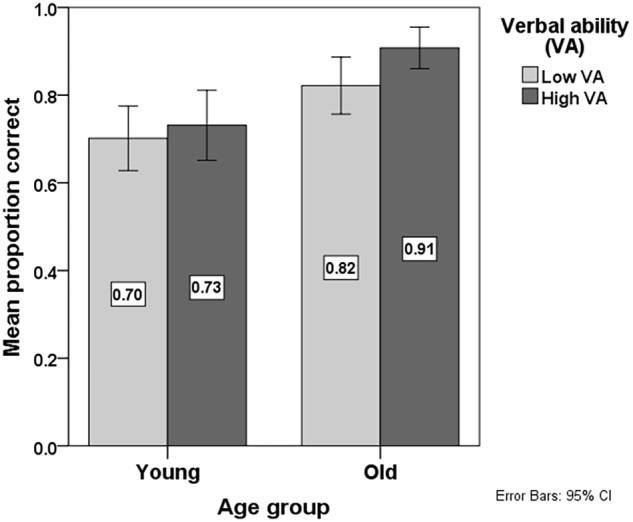
Mean proportion of correct responses in the language comprehension task per Age group (Young: ≤median vs. Old: >median; median = 9;3 years) and Verbal ability (VA) group (low VA: ≤median vs. High VA: >median; median = 108.50). Background information about the four groups plotted in this figure regarding their VA, as mean scores in the PPVT: Young-Low VA group: 99.86; Young-High VA group: 121.13; Old-Low VA group: 95.96; Old-High VA group: 118.48.

Finally, we checked for possible effects of the background variables IQ and VA on Accuracy. These analyses per predictor indicated main effects of IQ (*B* = 0.026; *SE* = 0.005; *p* < 0.001) and VA (*B* = 0.033; *SE* = 0.006; *p* < 0.001) and an interaction effect of VA^∗^Age (*B* = 0.001; *SE* = 0.00; *p* < 0.001). In model 3, we combined these main and interaction effects with the effects of model 2. Model 3 showed main effects of IQ and VA, indicating that children with a lower IQ and lower VA show a lower Accuracy than children with a higher IQ and higher VA, respectively. The interaction of VA^∗^Age remained significant in this analysis with multiple predictors, indicating that younger children (regardless of their VA), and older children with low VA, were less accurate in their interpretation of temporal conjunctions than older children with high VA, as is shown in **Figure 4**. Again, the median split method is used to plot Accuracy of temporal conjunction interpretation in each condition per VA group to illustrate the direction of the interaction effect. The figure caption of **Figure 4** provides background information about the VA performance of each group.

With the addition of IQ and VA, the main effect of WM disappeared (*p* > 0.05). The interaction effect of ToM2^∗^Congruency remained significant in model 3. Together, the results show that second-order ToM, WM, IQ, and VA play a role in the interpretation of temporal conjunctions. Individual and group differences therein explain why the TD group performs better than the ASD group and why older children perform better than younger children.

## Discussion

We investigated time perception in language by examining the interpretation of sentences containing the temporal conjunctions *before* and *after* by native Dutch school-aged children with and without ASD. We found, in line with our predictions, that children with ASD were less accurate than their TD peers at interpreting these temporal conjunctions. Contrary to our predictions, however, children with ASD did not have particular difficulties with temporal conjunctions in an incongruent compared to a congruent order. Furthermore, older children were found to perform better than younger children.

To understand the group and age effects, we examined which cognitive factors were associated with the interpretation of temporal conjunctions. Also, we examined whether the general background variables IQ and Verbal Ability affected interpretation. We found that age, IQ and VA were the major predictors of children’s correct interpretation of temporal conjunctions. Furthermore, the group effect was explained by differences in WM, second-order ToM understanding, IQ and VA. Children with ASD as well as TD children with lower WM made more errors when interpreting temporal conjunctions than children with higher WM. However, the effect of WM disappeared when taking into account children’s IQ. This is not surprising, given the strong relation between WM and IQ (Ackerman et al., 2005; Kidd, 2013). Also, IQ is a more broadly defined cognitive variable than WM and, in addition to measuring the simple short-term storage component of WM (Colom et al., 2008), also measures other cognitive abilities. VA appeared to underlie the age improvement in our study. Younger children, and older children with lower VA, made more errors when interpreting temporal conjunctions than older children with higher VA. This suggests that verbal skills must be sufficiently well developed for a mature understanding of complex sentences such as those involving temporal conjunctions. While suggested by previous studies (McCormack and Hanley, 2011; Blything et al., 2015), we found no effects of cognitive flexibility and cognitive inhibition (cf. de Ruiter et al., 2018). Particularly relevant for our research question and hypotheses was our finding that better second-order ToM understanding was positively associated with correct interpretation in an incongruent temporal order.

Although most children in our study showed a robust understanding of sentences containing temporal conjunctions, as predicted the children with ASD were less accurate than their TD peers at interpreting these sentences. In line with our hypotheses, this group difference between children with ASD and TD children was explained by differences in WM, second-order ToM understanding, IQ and VA. Because these cognitive functions are more likely to be impaired in children with ASD than in TD children (see Section “Introduction”), we attribute the poorer performance of children with ASD to their impaired cognitive functions rather than to their clinical diagnosis of ASD *per se*. Thus, our results actually suggest a much broader application than ASD, as the observed effects of cognitive factors on the interpretation of temporal language are likely to be relevant for typical development as well.

We did not find confirmation for our prediction that children with ASD have particular difficulties with temporal conjunctions in an incongruent order. Also, we did not find a main effect of congruency. The children in our study performed equally well on congruent as on incongruent items, in contrast to what has been found in several earlier studies with TD children (Clark, 1971; Trosborg, 1982; McCormack and Hanley, 2011; Pyykkönen and Järvikivi, 2012; Blything et al., 2015; de Ruiter et al., 2018). Possibly, we did not find a main effect of congruency because the children in our study were on average older (with a mean age of 9) than the children in most earlier studies and can be expected to have a more robust understanding of the meaning of the temporal conjunctions. Only one effect of congruency emerged from our data: children who make more errors in their interpretation of temporal conjunctions in an incongruent order were found to have a lower second-order ToM understanding. Good ToM understanding may thus help children to correctly interpret temporal conjunctions in an incongruent order, thereby suggesting that perspective taking is needed to interpret temporal conjunctions when the events are presented out of order.

One way to explain the role of ToM is that ToM understanding helps children to shift their perspective to another point in time in response to temporal language, and to understand the relationship between these different temporal perspectives on the same events (cf. McCormack and Hoerl, 1999; McCormack and Hanley, 2011). This explanation is in line with the literature on episodic memory based on the notion of mental time travel (Suddendorf and Corballis, 2007), or mental self-projection (Kretschmer-Trendowicz et al., 2016). Mental time travel involves a shift of the self from the immediate present to an alternative temporal perspective, for example, a past or future perspective (Buckner and Carroll, 2007; Suddendorf and Corballis, 2007). Several studies have suggested that there is a relation between mental time travel abilities and the comprehension of temporal language (Suddendorf and Corballis, 2007; Ferretti and Cosentino, 2013). In addition, it has been found that the neural processes involved in false-belief inferencing and the neural processes involved in mental time travel, in particular in taking the perspective of one’s future self to choose between an immediate and a future reward, overlap (O’Connell et al., 2018). In line with our results, this suggests that the comprehension of temporal language involves ToM understanding to enable hearers to shift from the immediate present to another point in time and perceive the situation from these different temporal perspectives.

An alternative possibility is that ToM understanding enables hearers to shift from their own perspective to the perspective of the speaker, for example, to find out why the speaker presented the events in an incongruent order. de Ruiter et al. (2018) explain their finding that children perform better with a congruent than an incongruent order in terms of the semantic principle of iconicity. They suggest that children initially assume an iconic (i.e., congruent) mapping between the order of events in the sentence and the order of events in the real world. Iconicity has been argued elsewhere to result from perspective taking; more complex, marked, forms tend to express more complex, marked, meanings (e.g., Horn, 1984; Levinson, 2000; Aissen, 2003). These more complex meanings have been argued to be acquired later in typical development than their less complex counterparts because they require the hearer to reason about why the speaker did not use the less complex form (e.g., De Hoop and Krämer, 2006; Hendriks et al., 2010). Incongruent meanings are more complex than congruent meanings. Also, sentences with *after* seem to be more complex than sentence with *before*, considering the *post hoc* effect of type of conjunction but not of clause order in our study (see note of **Table 2**) and the observation that *before* is acquired earlier than *after* (see Clark, 1971). Thus, a sentence with *after* may require the hearer to reason about why the speaker chose to use *after* rather than *before*, for example to foreground or background particular information. As mentioned above, our results indicate that children who make more errors in their interpretation of temporal conjunctions in an incongruent order have a lower second-order ToM understanding. Good ToM understanding may thus help hearers to correctly interpret temporal conjunctions in an incongruent order by allowing them to take the speaker’s perspective to find out why the speaker presented the events out of order.

In contrast to the study of de Ruiter et al. (2018), our study suggests that children need sufficient WM capacity for the interpretation of sentences containing temporal conjunctions. The different findings of the role of WM capacity could be the result of different WM measures. While we used a visuo-spatial WM task (an N-Back task) to operationalize WM capacity, de Ruiter and colleagues used three short-term memory tasks that do not require manipulation of the stored information (a word repetition task, a non-word repetition task and a sentence imitation task). These tasks may not have captured WM to the extent needed in complex sentence comprehension. Our findings confirm the results of Blything and Cain (2016), who used a verbal WM task (a digit span task) and also found a main effect of WM capacity on the interpretation of temporal conjunctions. Importantly, like Blything and Cain, we did not find that congruency interacted with WM in the accuracy task. This suggests that children’s difficulties with interpreting temporal conjunctions in an incongruent order are not explained by insufficient WM. Rather, children seem to need sufficient WM to process complex sentences conjoined by a temporal conjunction in general. These findings are corroborated by studies that have shown that individuals need WM capacity for the comprehension of other types of complex sentences as well, such as relative clauses and complement clauses (Just and Carpenter, 1992; Lewis et al., 2006; Montgomery et al., 2008; Boyle et al., 2013).

Turning to the implications of our study for ASD, previous research on temporal language in children with ASD mostly focused on production, showing deficits in the use of temporal adverbials and tense marking (Roberts et al., 2004; Colle et al., 2008). Here, we showed that verbal children with ASD also struggle with the interpretation of temporal conjunctions, due to weaker ToM understanding and lower WM capacity. This finding highlights the need to further study the interpretation of temporal expressions and temporal ordering in individuals with ASD. Languages have various ways to mark present, past and future and do so in almost every sentence. For example, English has tense marking on the finite verb, temporal adverbials such as *now*, *yesterday*, and *tomorrow*, and in addition to *before* and *after* also has other temporal conjunctions such as *when*, *while*, and *then*. A possibility for future research is to examine the interpretation of these and other temporal expressions in children with ASD. A second implication of our study for ASD concerns the nature of the language and communication difficulties in children with ASD. Linguistic deficits in verbal children with ASD are mostly viewed as difficulties with pragmatic aspects of language, which depend on its usage in context (American Psychiatric Association, 2013). However, the interpretation of temporal conjunctions depends on the meaning of the conjunction and its position in the sentence independently of their usage in context, and therefore, difficulty with their interpretation is structural (i.e., syntactic and semantic) rather than pragmatic in nature. In line with previous studies (Boucher, 2012; Durrleman et al., 2015), our results indicate the need to investigate the linguistic deficits in verbal children with ASD beyond pragmatics.

Summarizing, our study showed that children with ASD were less accurate at interpreting sentences containing temporal conjunctions than their TD peers, but did not have more difficulty in an incongruent rather than a congruent order. The different overall performance of children with ASD and TD children was explained by differences in second-order ToM understanding, WM, IQ, and VA, indicating that these factors likely contribute to the mature interpretation of temporal conjunctions. Specifically, second-order ToM understanding was associated with the interpretation of temporal conjunctions in an incongruent order, suggesting that perspective taking is needed to either shift one’s own perspective as a hearer from the immediate present to another point in time and relate these different temporal perspectives on the same events, or to shift to the perspective of the speaker to consider the speaker’s linguistic choices.

## Author Contributions

JO, CH, and PH contributed to the conception and design of the study. JO carried out the experiments. JO, CH, and PH analyzed the data. JO wrote the first draft of the manuscript. CH and PH wrote sections of the manuscript. All authors contributed to manuscript revision, read, and approved the submitted version.

## Conflict of Interest Statement

The authors declare that the research was conducted in the absence of any commercial or financial relationships that could be construed as a potential conflict of interest.
